# Accidental chest penetration of a glass foreign body

**DOI:** 10.36416/1806-3756/e20250211

**Published:** 2025-09-22

**Authors:** Simone Duarte Damato, Alessandro Severo Alves de Melo, Edson Marchiori

**Affiliations:** 1. Universidade Federal Fluminense, Niterói (RJ) Brasil.; 2. Universidade Federal do Rio de Janeiro, Rio de Janeiro (RJ) Brasil.

**Figure 1:**
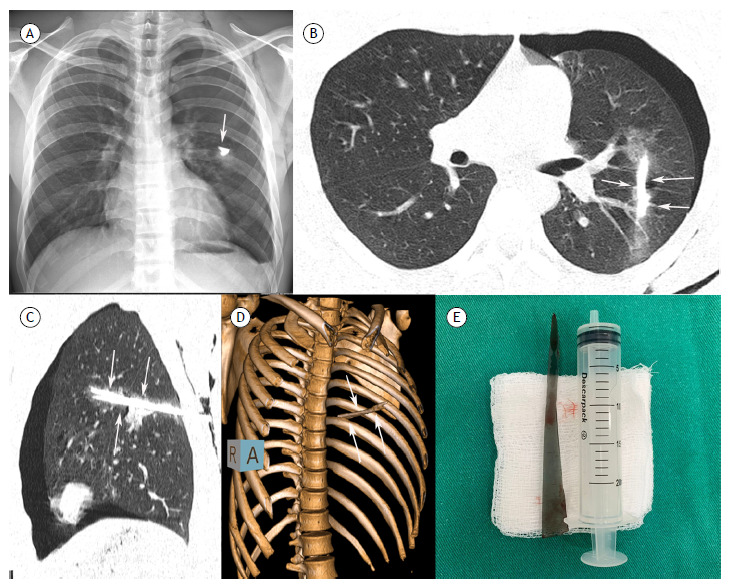
Chest radiograph (A) showing a dense foreign body in the left hemithorax (arrow). Axial (B) and sagittal (C) chest CT images and 3D reconstruction (D) demonstrating a dense linear foreign body in the left lung and ground-glass opacities (hemorrhage) in the periphery. Note also the pneumothorax on the left. (E) The mirror fragment removed from the patient, measuring about 30 cm.

A 20-year-old previously healthy man reported falling from his own height onto a mirror and suffering a cut in the left infrascapular region. He sought emergency care, where the cut was sutured. He continued to have pain in the area and returned the following day, when a chest X-ray showed a foreign body on the left (Figure 1A). The patient was referred to the hospital, where further imaging showed a high-density linear foreign body in the left hemithorax, in addition to a pneumothorax (Figure 1B-E). He underwent pleural drainage and surgery, during which a glass foreign body (mirror fragment) measuring approximately 30 cm was removed (Figure 1E). The patient progressed well and was discharged four days later in excellent condition.

Intrathoracic foreign bodies include iatrogenic foreign bodies, objects that have migrated through the airways, and traumatic intrathoracic foreign bodies. Glass exhibits high density on chest radiography and CT. CT is the best imaging method for the evaluation of such trauma. Even minor impalement injuries may cause serious complications, including organ damage and life-threatening bleeding. Accurate visual, manual, and instrumental wound exploration is always necessary. Surgical removal of the foreign body is the first-choice treatment.^1^ ^,^ ^2^
